# Coinfection with dengue and hepatitis A complicated with infective endocarditis in a Yemeni patient: a case report

**DOI:** 10.1186/s13256-021-03069-w

**Published:** 2021-09-11

**Authors:** Shafiq A. Alemad, Abdulsalam M. Halboup, Khaled Aladeeb, Mohamed Al-Saleh, Nuha Al-Kufiley

**Affiliations:** 1 University of Science and Technology Hospital, Sana’a, Yemen; 2 Departments of Clinical Pharmacy and Pharmacy Practice, Faculty of Pharmacy, University of Science and Technology, P.O. Box 13064, Sana’a, Yemen

**Keywords:** Infective endocarditis, Dengue, Hepatitis A, Coagulase-negative staphylococci, Yemen

## Abstract

**Introduction:**

Coinfection with dengue and hepatitis A is rare and challenging for physicians since their clinical features can be overlapping. These infections are self-limiting but can become complicated by subsequent infective endocarditis. We report a case of infective endocarditis following a coinfection with dengue and hepatitis A.

**Case presentation:**

A 17-year-old Yemeni male patient was admitted to the hospital complaining of yellowish discoloration of the skin and sclera associated with dark urine and a diffuse skin rash on the trunk and upper limbs followed by intermittent high-grade fever. Coinfection was confirmed by hepatitis A immunoglobulin M and dengue immunoglobulin M. At the time of diagnosis, white blood cells were normal, with mild neutrophilia and thrombocytopenia along with elevated C-reactive protein. Five days later, the patient was readmitted to the emergency department, complaining of high-grade fever, fatigue, myalgia, nausea, and vomiting. A systolic heart murmur was heard, and infective endocarditis was confirmed by the visualization of two vegetations on the mitral valve and coagulase-negative staphylococci after blood culture. Supportive therapies were initiated for hepatitis A and dengue fever, whereas infective endocarditis was treated with antibiotics for 4 weeks. The patient recovered completely from dengue, hepatitis A, and infective endocarditis.

**Conclusion:**

In endemic areas, it is reasonable to screen for coinfection with dengue and hepatitis A since they are superimposed on each other. Subacute infective endocarditis may occur following initial dengue and hepatitis A coinfection, especially among patients with rheumatic heart disease. An echocardiogram is a pivotal workup for evaluating a patient with persistent fever of unknown origin.

**Supplementary Information:**

The online version contains supplementary material available at https://doi.org/10.1186/s13256-021-03069-w.

## Introduction

Dengue is a public health concern in more than 100 countries worldwide [1]. According to World Health Organization (WHO) guidelines, dengue severity is classified into dengue with/without warning signs and severe dengue [2]. Dengue is endemic in Yemen, with reports on several outbreaks in Hodeidah, Taiz, Aden, and Hadhramout governorates [3–6]. People displacement, poor hygiene, and damaged health infrastructure due to the civil war are the main factors contributing to the spread of mosquito-borne diseases in the country [6, 7].

Coinfection with dengue is frequently caused by bacteria, followed by viruses [8]. There are several case reports on bacterial infections causing complications in patients with dengue. For instance, dengue can be complicated by *Staphylococcus aureus* causing pneumonia [9, 10] and abscess [11–13]. Additionally, staphylococcal endocarditis following classic and hemorrhagic dengue fever was also reported [14, 15].

Coinfection with dengue and hepatitis A in an individual is rare but challenging for medical professionals because of the overlap between their symptoms [16, 17]. Moreover, bacteremia in a patient coinfected with dengue and hepatitis A is extremely rare and more challenging in terms of diagnosis and management, posing life-threatening complications. We report a case of an adult male who was initially diagnosed with dengue and hepatitis A coinfection but subsequently developed infective endocarditis by coagulase-negative staphylococci (CoNS).

### Case presentation

A 17-year-old Yemeni male patient with rheumatic heart disease and a travel history to Hadhramout governorate in the east of the country was admitted to the hospital complaining of yellowish discoloration of the sclera and skin along with dark urine but without dysuria or frequency. Then, he developed an intermittent high-grade fever, headache, fatigue, myalgia, and abdominal discomfort together with food-related nausea, vomiting, and loss of appetite. On examination, the patient was febrile and had yellowish discoloration of the sclera along with a skin rash on his trunk and upper limbs. Systemic examination revealed bilateral basal lung crepitation along with mild tenderness and hepatomegaly, while cardiovascular and other systems were initially normal. The patient underwent tonsillectomy 10 years earlier and was on benzathine penicillin 6 months before admission. Socially, the patient was not a smoker, alcoholic, or drug-addicted.

The investigations performed on presentation (3 July 2019) to the Emergency Department revealed a normal white blood cell (WBC) count of 10.6 × 10^9^/L (normal range: 4–11 × 10^9^/l), with a slightly increased neutrophil percentage to 83% (normal range 40–75%). Serum C-reactive protein (CRP) was 253 mg/L (normal level < 5 mg/L). The platelet count decreased to 115 × 10^9^/L but then gradually increased. Peripheral blood smear examination showed normocytic, normochromic red blood cells (RBCs) with normal morphology of all cell types. Serum albumin concentration was 27.4 g/L (normal 35–45 g/L), and urine was positive for bilirubin but did not contain RBCs and pus cells. Alanine aminotransferase level was 401 U/L (normal < 50 U/L), while aspartate aminotransferase, alkaline phosphatase, and serum creatinine were within the normal range. Direct bilirubin concentration was 6.6 mg/dL (normal < 0.3 mg/dL) and increased to 8.96 mg/dL on the readmission day (8 July 2019) but then decreased gradually to 1.65 mg/dL on discharge (17 July 2019). International normalized ratio and prothrombin time were 1.69 and 20.4 seconds (normal 13.5 seconds), respectively. The other investigations done during clinic visits, readmission, and before discharge are summarized in Table 1.

**Table 1:** Investigation details of the reported case

Parameters (test)	Findings on first admission (3/7/2019)	Finding after discharge (6/7/2019)	Finding on readmission (8/7/2019)	Findings on first visit (20/7/2019)	Findings on second visit (27/7/2019)	Normal value
Hemoglobin (g/dL)	13.6	14.6	11.9	11.0	10.7	**13–18**
WBC (cells/mm^3^)	10.6	10.1	21.77	5.9	6.4	**4–11 × 10**^**9**^**/L**
Neutrophil	83	69	93.4	53	50.0	**40–70%**
Lymphocyte	12	26	3.2	42	45.9	**20–45**
Platelets (cells/mm^3^)	115	135	168	432	377	**150–450**
Total bilirubin (mg/dL)		11.11	9.843			**0.1–1.2 mg/dL**
Direct bilirubin(mg/dL)	6.6	10.7	8.96	1.92	1.22	**< 0.3**
SGOT (AST)	40	44.0	–	–	–	**< 50**
SGPT (ALT)	401	198	154	43	43.0	**Male < 50**
Alkaline Phosphatase (U/L)	144	268	–	–	–	**Male < 270**
Hepatitis B surface antigen (HBSAg)	Negative	–	–	–	–	**Negative < 1**
C-reactive protein (CRP) by immunoturbidimetry	253	14.0	67	3.92	1.45	**Negative < 5 mg/L**
ESR (mm/hour)	–		–	–	50	**Up to 10 mm/hour**
Serum albumin (g/L)	27.4	31.9	–	39.9	–	**35–45**
Total protein (g/L)		80.3				**Adult: 68–87**
Serum creatinine (μmol/L)	78.69			124	110	**Adult: < 106**
Dengue virus IgM by (ELISA)	16	–	–	–	–	**Negative < 9**
Hepatitis A IgM antibody	11.66	–	–	–	–	**Negative < 0.8**
Brucella total antibody titer by agglutination	Negative	–	–	–	–	**< 1:80**
Prothrombin time (seconds)	20.4	19.0	18.3	13.8	–	**Control: 13.5**
International normalized ratio (INR)	1.69	1.51	1.47	1.06	–	**–**
Malaria test	Blood smear: no malaria seen Rapid test (antigen): negative	–	–	–	–	–

Bold font represents normal laboratory values

*WBC* white blood cells, *ALT* alanine aminotransferase, *AST* aspartate aminotransferase, *ESR* erythrocyte sedimentation rate, *ELISA* enzyme-linked immunosorbent assay

The clinical diagnosis of acute liver injury as a result of possible dengue and hepatitis coinfection was made based on the travel history to Hadhramout governorate, clinical presentation, and clinical examination. Both viral infections were confirmed by dengue immunoglobulin M (IgM) and hepatitis A IgM antibodies test using enzyme-linked immunosorbent assay (16 and 11.66; negative results for dengue and hepatitis A are less than 9 and 0.8, respectively) on admission (3 July 2019). Tests for malaria and brucellosis were negative.

In the emergency department, supportive therapies for both hepatitis A and dengue were commenced using intravenous fluids, antipyretics, and antiemetic. Then, the patient was discharged home on intravenous dextrose saline, paracetamol 1 g four times a day as needed, and azithromycin 500 mg once daily for 3 days. Azithromycin was given for suspected bacterial infection.

Five days later, the patient was readmitted (8 July 2019, the first day of readmission) to the Medical Male Ward complaining of high-grade fever not relieved by simple antipyretics associated with nausea and vomiting, malaise, myalgia, and decreased appetite. His heart rate and blood pressure were 112 beats/minute and 100/60 mmHg, respectively. The total WBC count increased to 21.77 × 10^9^ /L, with 93.4% of the cells being neutrophils, while CRP was 67 mg/L on the first day and increased to 140 mg/L on the second day of illness.

**Fig. 1:**
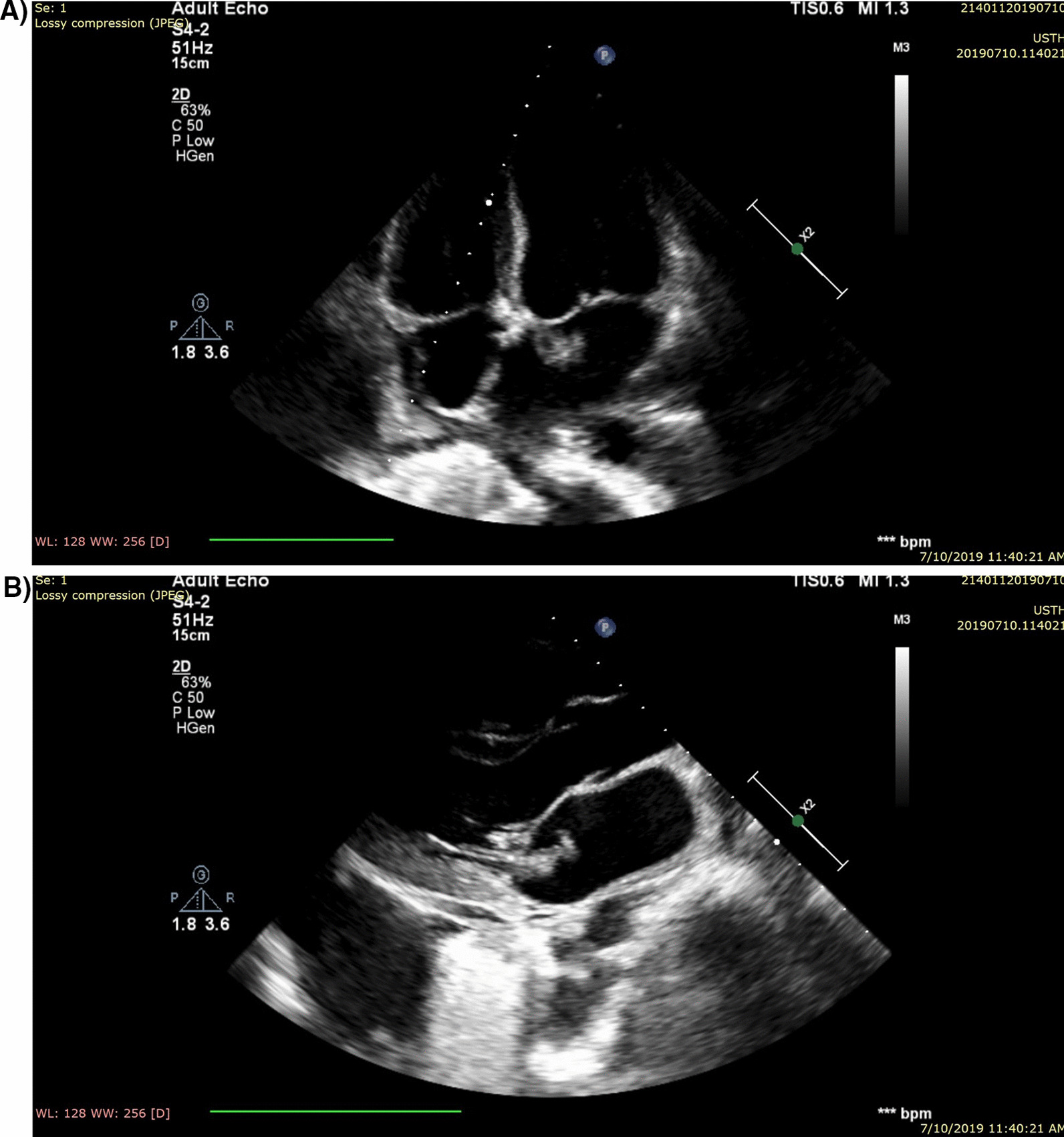
Echocardiogram showing two mitral valve vegetations. **A** Anterior leaflet vegetation of the mitral valve measuring 24 × 11 mm. **B** Posterior leaflet vegetation of the mitral valve measuring 2.1 × 0.9 cm

Parenteral empirical therapy using meropenem (500 mg three times a day) with moxifloxacin (400 mg once a day) was started on the readmission day, and vancomycin (1 g twice a day) was added on the third day (10 July 2019) after a noticeable increase in the CRP levels. Further clinical examinations and laboratory investigations were performed to identify the source of infection. On examination, there was a systolic murmur (grade 3) at the mitral area and sacral edema on his back. The patient's chest echocardiogram revealed moderate mitral regurgitation and two vegetations on the mitral valve. The large one (24 × 11 mm) attached to the anterior leaflet of the mitral valve, while the small one (2.1 × 0.9 cm) attached to the posterior leaflet of the mitral valve as shown in Fig.1A, B. There was also mild tricuspid regurgitation with moderate pulmonary hypertension. The estimated ejection fraction by M-mode was 72%. Other valves and the dimensions of the cardiac chambers were normal with no pericardial effusion. On the same day, the chest echocardiogram was repeated and showed the same findings as the first day. According to the modified Duke criteria, the diagnosis of infective endocarditis was made based on one major criterion (presence of vegetation) and two minor criteria (single positive culture and fever). On the fourth day of hospitalization, a single blood culture yielded CoNS sensitive to ampicillin–sulbactam, cefoperazone–sulbactam, ceftriaxone–tazobactam, ciprofloxacin, clindamycin, doxycycline, gentamicin, imipenem, levofloxacin, lincomycin, moxifloxacin, and vancomycin, but it was resistant to amoxicillin/clavulanic acid and co-trimoxazole. At this time, meropenem and moxifloxacin were discontinued, and the patient was treated with ceftriaxone/sulbactam (1.5 g twice a day for 4 weeks) with a daily dose of gentamicin (240 mg) with 100 ml normal saline over 1 hour for 2 weeks.

On the seventh day of hospitalization, the patient complained of diarrhea (four times per day) for which upper abdominal ultrasound and *Clostridium difficile* antigen were requested. Ultrasound showed moderate hepatomegaly (16 cm) with mild bilateral pleural effusion, while *C. difficile* antigen test was negative. Therefore, the treatment plan was not modified.

Treatment progress was monitored through the daily investigation of CRP, WBCs, and alanine aminotransferase. The patient was discharged on the tenth day of hospitalization after revealing a marked improvement on clinical and laboratory investigations. Other medications were added to the above-mentioned antibiotics, including bisoprolol (5 mg tablet once daily for 1 month) to control tachycardia, paracetamol (500 mg tablet for fever) as needed, furosemide (40 mg tablet once daily in the morning for 1 month) along with metolazone (1 mg tablet for 10 days) for pleural effusion and sacral edema. No additional echocardiogram was performed after discharge. During the first and second follow-up visits to the medical clinic, on day 3 and day 10 after discharge, the clinical features and laboratory investigations of the patients showed obvious improvement as summarized in Table 1.

### Discussion

We report a case of infective endocarditis diagnosed a few days after the diagnosis and management of coinfection with dengue and hepatitis A in a 17-year-old Yemeni male patient. Although dengue and hepatitis A were diagnosed before infective endocarditis, we assumed that the latter coexisted with both infections but remained undiagnosed. This hypothesis is in agreement with the fact that our patient had a risk factor (rheumatic heart disease), was subjected to tonsillectomy 10 years earlier, and was on benzathine penicillin G (1.2 million units every 3 weeks) until 6 months before admission. The diagnosis of infective endocarditis was not established at the time of dengue and hepatitis A diagnosis because of several factors. These include the inability to perform blood cultures and echocardiogram at the first admission of viral coinfection since the patient was admitted and discharged home from the emergency department on the same day based on his request. Additionally, the long delay in the diagnosis of CoNS-induced infective endocarditis could be related to its indolent, mild, and prolonged disease course [18, 19]. Furthermore, infective endocarditis is easily overlooked in a patient coinfected with dengue and viral hepatitis because of the overlap between several clinical features of such diseases that can pose a diagnostic dilemma. Triple infections are also sparsely reported in the literature.

Bacterial infections are the most frequent type of dengue-associated coinfection. Several mechanisms have been proposed to explain the increased susceptibility to secondary bacterial infection following dengue. One mechanism is the damage to the endothelial tissue of the endocardium, predisposing to bacteria colonization and endocarditis during skin breaching by cannula [20]. Another mechanism is the dysregulation of the immune system caused by high levels of interleukin-10 among dengue patients, predisposing them to secondary bacterial infection [21].

Several studies have described multiple infections in patients with dengue. Patients with severe dengue [10], prolonged fever (more than 5 days), with or without acute renal failure [22], increased WBC count, decreased lymphocytes [23], or hyponatremia [24] are at high risk for concurrent bacterial infection. Most of these factors predispose a patient to develop infective endocarditis. In our case, several risk factors, including prolonged fever, leukocytosis, decreased lymphocytes, increased CRP, high pulse rate, and hyponatremia along with hepatic failure, predispose him to develop infective endocarditis.

Staphylococcal infections following classic or hemorrhagic dengue were reported in patients with thyroid abscess [11], intramuscular abscesses [12, 13], infective endocarditis [14, 15, 25], and pneumonia [9, 10]. Furthermore, CoNS were isolated from hospitalized adults with dengue [26]. In our case, skin breaches occurring during cannulation might have facilitated the entry of CoNS into his blood circulation, leading to bacteremia and subsequent infective endocarditis (Additional file 1, Additional file 2).

Coinfection with dengue and hepatitis A has been reported in several case reports [16, 17, 27–30]. Overlapping of clinical features between dengue and hepatitis A can lead to substantial misdiagnosis. Therefore, it is reasonable to screen for both types of infection in endemic areas. In our case, the marked increase in serum aminotransferase levels, deranged prothrombin time, and jaundice were common features of acute viral hepatitis, while thrombocytopenia, hemoconcentration, hepatomegaly, abdominal tenderness, and third spacing were common features of dengue with warning signs.

### Conclusion

In endemic areas, coinfection with dengue and viral hepatitis can be encountered among young adults with rheumatic heart disease. A patient presenting with elevated liver enzymes, deranged coagulation profile, prolonged fever, pleural effusion, and thrombocytopenia should alert clinicians towards dengue and hepatitis A coinfection. Highly elevated aminotransferase levels (eight to ten times the upper limit) are consistent with acute viral hepatitis compared with two to three times in dengue fever, and the ratio of aspartate aminotransferase to lactate dehydrogenase is more than 4 in acute hepatitis. Nevertheless, elevated CRP and high pulse rate along with prolonged and high-grade fever in a patient with dengue should be considered as alarming signs for concurrent bacterial infection. Therefore, echocardiography and blood culturing are pivotal workups in patients with persistent fever of unknown origin. Moreover, timely administration of optimal antibiotics is critical for patients with mixed infections to avoid life-threatening complications.

This study has some limitations. One of the limitations is that the specific pathogen of CoNS that induced endocarditis in this patient was not determined due to a resource-limited setting. The second important limitation is that blood samples for cultures were collected after the administration of empirical antibiotics. One more limitation is that several laboratory investigations were not requested during hospitalization since the patient was not covered by health insurance.

## Supplementary Information

**Additional file 1.** The video shows anterior leaflet vegetation.

**Additional file 2.** The video shows posterior leaflet vegetation.

## Data Availability

The data that support the findings of this case report are available from the medical records of the male medical department at University of Science and Technology Hospital, Sana’a, Yemen.

## Abbreviations

CoNS: Coagulase-negative staphylococci
CRP: C-reactive protein
WBC: White blood cells
RBCs: Red blood cells
